# Citizen scientists filling knowledge gaps of phosphate pollution dynamics in rural areas

**DOI:** 10.1007/s10661-024-12389-5

**Published:** 2024-01-31

**Authors:** Steven Loiselle, Isabel Bishop, Heather Moorhouse, Caroline Pilat, Eline Koelman, Rosie Nelson, Wim Clymans, John Pratt, Vaughn Lewis

**Affiliations:** 1 Earthwatch Europe, Mayfield House, 256 Banbury Road, Oxford, OX2 7DE UK; 2 University of Siena, INSTM, 53400 Siena, Italy; 3 Centre for Biodiversity and Environment Research (CBER) UCL, London, WC1H 0AG UK; 4 UK Centre for Ecology and Hydrology, Library Avenue, Bailrigg, Lancaster LA1 4AP UK; 5 ARUP, Ove Arup & Partners Ltd, 4 Pierhead Street, CF10 4QP Cardiff, UK; 6 Thames Water, Clearwater Court, Vastern Road, Reading, RG1 8DB UK; 7 Flemish Institute for Technological Research, Boeretang 200, 2400 Mol, Belgium; 8 Coldstone Angling Club, Ascott-Under-Wychwood, OX7 6AF UK; 9 Windrush AEC Ltd, Fordwells, UK

**Keywords:** Nutrient pollution, Citizen science, Eutrophication, Stream monitoring

## Abstract

In situ monitoring is fundamental to manage eutrophication in rivers and streams. However, in recent decades, the frequency and spatial coverage of regulatory monitoring have often been reduced due to funding and infrastructure limitations. This reduction has made it impossible to provide adequate coverage for most water bodies. In this study, trained citizen scientists filled spatial and temporal gaps in agency monitoring across a major catchment in rural England. By integrating data from citizen scientists, regulatory agencies, and the local water company, it was possible to demonstrate the opportunities for hypothesis-based citizen scientist monitoring to identify continuous and event-driven sources of phosphate pollution. Local citizen scientists effectively covered important spatial gaps, investigating river conditions both upstream and downstream of suspected pollution point sources, improving the identification of their temporal dynamics. When combined with long-term monitoring data from regulatory agencies, it became possible to identify areas within the catchment that exhibited increased phosphate concentrations during periods of low river discharge (summer). Inter-annual trends and anomaly detection suggested that continuous pollution sources dominated over event-driven sources in many sub-basins, allowing for the prioritisation of mitigation actions. This study highlights the opportunity for citizen scientists to fill gaps in regulatory monitoring efforts and contribute to the improved management of eutrophication in rural catchments.

**Supplementary Information:**

The online version contains supplementary material available at 10.1007/s10661-024-12389-5.

## Introduction

Eutrophication or excessive nutrient enrichment continues to be one of the major threats to the ecology of rivers and lakes across the world (Ansari et al., 2010). Over the last century, there has been a generalised increase in nutrient concentrations, usually associated with expanding population centres, land use and land cover change (Walsh et al., 2005). Efforts in recent decades to control and reduce the input of phosphorus and nitrogen through improved wastewater treatment and more integrated agricultural nutrient management have shown some success in restoring eutrophic ecosystems (Smith & Schindler, 2009). Phosphorus, in particular, plays a key role in eutrophication in river systems in southern UK (Hutchins and Hitt, 2019). Mitigation of diffuse and direct phosphorus loads has been associated with improved conditions in lakes and rivers. In Europe, the Water Framework Directive and the European Union Urban Wastewater Treatment Directive have led to an improved conditions in many waterbodies, but the overall progress to achieving good chemical status of European rivers is limited (Pistocchi et al., 2019; Zacharias et al., 2020).

Phosphorus is typically a limiting nutrient in most unimpacted freshwater ecosystems. When present in high concentrations, phosphate favours the formation of harmful algal blooms as well as an increase in epiphytic and benthic algae (Jarvie et al., 2006; Mallin & Cahoon, 2020; O’Hare et al., 2018). These changes can affect the growth rate and composition of macrophytes, with important impacts on the fish population and sensitive animal species, strongly impacting biodiversity in rivers (Mainstone & Parr, 2002). Elevated phosphate concentrations are a key driver of the river degradation in England and have been identified as a priority for the rivers and streams in the Evenlode catchment, the focus area of this study (Hutchins and Hitt, 2019; Jarvie et al., 2006). These negative aspects are further compounded by increases in the concentration of fine particulates, with consequences on river habitat (Amorim and do Nascimento, 2021). Excessive algal growth can lead to hypoxic ‘dead zones’ that reduce fish populations as well as generate compounds that impact the safety of water supplies and human use of the waterbody.

Anthropogenic phosphorus sources can have multiple pathways, which can change throughout the year, in relation to weather (precipitation and temperature) and human activities. The impact of increased phosphate concentrations will depend on hydrological (e.g. river discharge) and ecological (e.g. macrophyte communities) conditions that change over time and space. Most catchments have multiple phosphorus sources, those most important being wastewater discharges, both event-driven and continuous, agricultural emissions (including livestock, pasture and forestry), industrial effluents and runoff from impervious surfaces (roads) as well as natural sources related to local geology. In agricultural areas, mineral fertiliser and manure are used to provide phosphorus to improve plant growth. However, a large portion of the applied phosphorus is not taken up and accumulates in the soil (Syers et al., 2008). This excess phosphorus enters surface water through precipitation-driven erosion, while subsurface flow may be important in intensively drained agricultural settings (King et al., 2015).

A major source of phosphorus in urban and rural areas is treated or untreated wastewater. Differently from diffuse nutrient loads, human wastewater emissions are typically point source emitters (Rabalais et al., 2010). While the overall water quality has improved in many urban areas, rivers in rural areas have often been left behind, with a continuing decrease in water quality and limited attention from authorities (Whelan et al., 2022). Untreated wastewater contains large nutrient loads from human and animal excreta (Billen et al., 2012; Li et al., 2012). Van Dijk et al. (2016) showed that, of the 2400 Gg of phosphorus imported into Europe in 2005, more than half was lost as waste, with consumption-based waste flows dominating. Of these latter, the largest losses (655 Gg) were wastewater (55%), followed by food waste (27%), and pet excreta (11%). The concentrations of phosphorus in the discharge from sewage treatment works (STWs) depend on population, STW capacity and level of treatment.

Whilst nutrient pollution is a major challenge to maintaining functioning freshwater environments, monitoring of these conditions is often limited, due to poor design, by a lack of funding and political directives (Fones et al., 2020; Lindenmayer & Likens, 2010; Lovett et al., 2007). Even when appropriately funded, regulatory monitoring may not deliver effective monitoring to cover the spatial and temporal variability of nutrient concentrations present in most catchments (Varekar et al., 2021). With the advent and the expansion of alternative monitoring approaches, from remote sensing to citizen science, there are new opportunities to improve spatial and temporal monitoring of rivers or lakes to better understand and mitigate pollution sources (Collins et al., 2023). Numerous studies have shown that citizen science data can help identify pollution drivers and prioritize action (Loiselle et al., 2016; Thornhill et al., 2018). A central challenge remains the integration of data from these approaches to regulatory monitoring, which are often undertaken at different spatial and temporal scales and may use different analytical approaches.

**Fig. 1:**
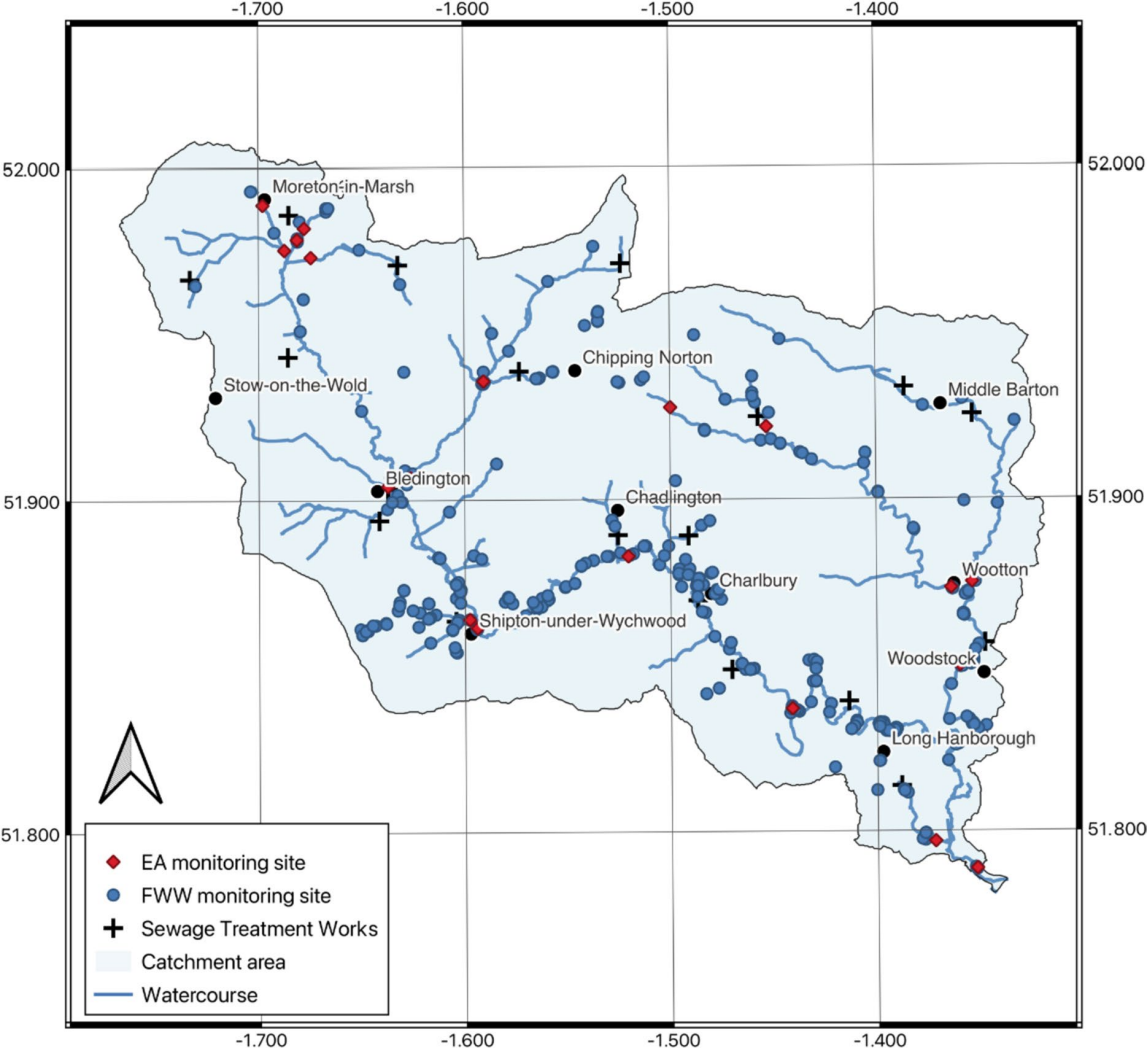
Evenlode catchment in Oxfordshire, South East England (UK), with the sites of regular monitoring performed by the national regulatory agency (Environment Agency (EA), 20 sites with at least 100 monitoring events), citizen science (FreshWater Watch (FWW)) monitoring sites as well as the location of sewage treatment works

The present study is focused on the use of measurements made by trained citizen scientists to fill spatial and temporal gaps in regulatory monitoring of orthophosphate, in a large temperate river catchment. The Evenlode catchment (430 km^2^) contains 18 river waterbodies, with the major tributaries being the Glyme and the Dorn (Fig. 1). It is an important affluent to the Thames River. The catchment is characterised by beechwoods and limestone grasslands, lowland meadows and fen, all of which support a wide range of wildlife. The river habitat in the Evenlode catchment has been compromised by a combination of historical channel modification, with land use dominated by agriculture, particularly arable farming. The area is predominantly rural, with small towns present throughout the catchment, the largest being Moreton-in-the-Marsh and Charlbury, both with populations below 3500 inhabitants. Population growth in the area averages 1% per year, since 2011. The catchment contains several lakes and landscape areas with important national and international significance: Sites of Special Scientific Interest, Conservation Target Areas and World Heritage sites. The catchment has multiple phosphorus sources, with treated and partially treated wastewater and agricultural activities being the most dominant. Most STW are of small-to-medium dimension and do not have tertiary treatment, where phosphorus and nitrogen contained in the wastewater are typically removed using a range of chemical and physical processes (Bunce et al., 2018).

In response to degrading conditions of the catchment, the Evenlode Catchment Partnership was formed in 2014, bringing together key stakeholders, including the country wildlife charity (Wild Oxfordshire), the local water company (Thames Water), the national regulatory agency (Environmental Agency, EA), an environmental charity (Earthwatch Europe), local consultants, angling organisations, riparian landowners and, most importantly, the local communities. The partnership was initially funded by local grants and is partially funded by the Thames Water Smarter Water Catchments initiative. The collaborative partnership brings together local and national knowledge and aims to improve the water environment across the catchment in the short term (i.e. between 2020 and 2025).

### Multilayer monitoring

**Fig. 2:**
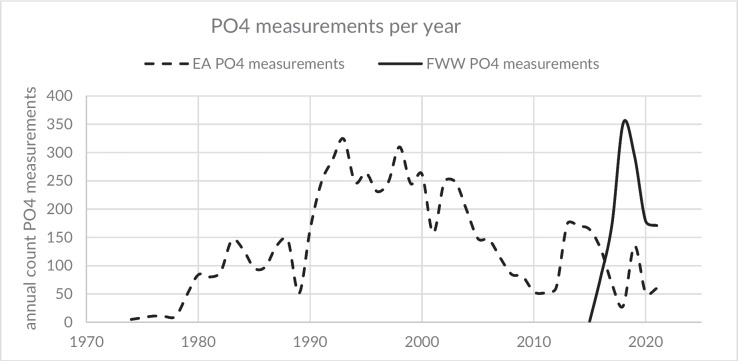
Number of annual monitoring events by the national regulatory agency (Environment Agency (EA) and by citizen scientists (FWW) of phosphorus phosphate (PO4) in the Evenlode catchment (all sites)

This study combines phosphate concentration data from trained citizen scientists and regulatory EA monitoring, together with Thames Water sewage treatment effluent monitoring. The integration of data from multiple sources, providing different temporal and spatial coverage, improves understanding of pollution sources and the impact of mitigation actions. Spatial and temporal data gaps in monitoring coverage can be reduced when data from sources from alternative to regulatory agencies are considered. With the advent of robust citizen science programmes, this source of complementary monitoring data provides an important opportunity to improve catchment management. In the study catchment, the annual frequency of monitoring events performed by the EA has varied significantly over the last five decades, with the maximum number of annual monitoring events occurring in the 1990s and the minimum occurring in 2018 (Fig. 2). This has largely followed changes in national monitoring strategies, as well as changes in local monitoring priorities. Impacts related to international drivers (e.g. COVID-19 restrictions) are also evident. Citizen scientists have been monitoring in the catchment since 2016, using the global FreshWater Watch (FWW) platform and standard methods. It should be noted that the measurement frequency of citizen scientists is higher than that of the EA since 2017. The citizen scientist monitoring activities were and are intended to fill temporal and spatial gaps as well as identify areas of concern to support a better understanding of the catchment. Since 2018, the EA has monitored regularly 14 locations in the catchment, while citizen scientists have monitored 21 locations regularly and nearly 100 on a less regular basis. Spatially, most sites monitored by the EA cover areas downstream of potential pollution areas as well as the main channel of the Evenlode river (Fig. 1). Citizen scientists have largely focused on the river tributaries. Interestingly, many citizen scientists adopted a hypothesis-driven approach, performing regular monthly monitoring in both upstream and downstream sites of suspected pollution point sources over the course of several years. This fills gaps of regulatory monitoring as well as providing direct information on the impact of active pollution sources.

In the present study, we explore how combining data from citizen scientists, regulatory agencies (EA) and industrial stakeholders (Thames Water) can allow for a more complete understanding of the pollution drivers and water quality conditions of a major catchment.

## Methods

EA determine orthophosphate concentrations (PO_4_) using ammonium molybdate and antimony potassium tartrate under acidic conditions and determining absorbance at 880 nm using a standard spectrophotometer, following calibration. Historical EA data on PO_4_ concentrations were available from 1974 to 2021, with a total of 6500 measurements (Table S1). Monitoring frequency averages 6.5 events per year per location. EA monitoring is performed mostly on weekdays and typically in a single location in the lower reaches of individual waterbodies. A total of 20 sites, with a minimum coverage over the last two decades, were used in the present analysis of seasonal and trend analysis (Fig. 1). Citizen scientists have been monitoring PO_4_ concentrations alongside nitrate concentrations and turbidity using a standardised monitoring kit (FWW) (Thornhill et al., 2018). All citizen scientists have followed a consistent in-person training in the field which was supported by online training videos, automated feedback and in-person feedback in local meetings. As quality control begins with the citizen scientist, both the in-person field training and the continued interaction with the project leads were fundamental.

The FWW measurements of PO_4_ are made colourimetrically in closed tubes using a standard plastic cuvette for a fixed volume of 1.5-mL standard colour reference cards provided to each citizen scientist. PO_4_ is detected using 4-amino-antipyrine with phosphatase enzyme which produces a coloured solution which is compared to a standard reference colour chart, assigning colour brightness to one of seven concentration intervals. Side-by-side measurements have shown an overall accuracy of 75% to 85% of the citizen scientist estimated PO_4_ concentrations compared to concentrations measured at the same site and day by professional scientists using standard laboratory analysis (Hegarty et al., 2021; Moshi et al., 2022). Sources of errors include the misreading of the colorimetric scale and differences between the time of measurement, directly in the field for citizen scientists and after transportation to the lab for professional scientists. Comparisons between citizen scientists measuring the same sample have been performed in individual projects and shows high inter-rater agreement (Cohen’s κ > 0.70). Quality control of all data is performed by Earthwatch Europe and by local partners by identifying anomalies and inconsistencies within and between datasets (Moshi et al., 2022). Automated feedback is provided through the app at the moment of data acquisition to the citizen scientist, based on inconsistencies within each completed dataset. Regular feedback regarding quality control is also provided directly by local project leaders as well as by Earthwatch Europe following monthly data checks and considering past measurements made in the same location as well as local knowledge. Quality control of kit reagents is performed regularly in partner laboratories.

Since 2015, there have been 1800 citizen science monitoring events in the catchment, of which 950 occurred during annual catchment blitz events, where hundreds of trained citizen scientists monitor catchment conditions over a single weekend (Hadj-Hammou et al., 2017). These measurements cover nearly 700 sample locations, with 21 locations being regularly monitored by citizen scientists, as part of a monthly monitoring programme which began in 2018. The average frequency of those sites selected for regular monitoring by citizen scientists is 9.5 events per year. The selection of measurement sites is made by citizen scientists, following training and with the support of local partners, to be complementary to regulatory monitoring, covering areas no longer monitored or areas that lack monitoring. In recent years, their selection has focused on locations that are not regularly monitored by EA scientists, taking measurements above and below suspected pollution sources. Monthly concentrations obtained on the same day by citizen scientists from sites located above and below suspected pollution sources were compared to identify monthly variations in concentrations related to suspected sources and related impact on receiving rivers. These data were also compared using Pearson correlations to explore the influence of the upstream conditions on the downstream site, with respect to the pollution source.

It should be noted that site selection is also influenced by access and proximity to their residence or working place. An additional selection bias is related to the day of the week of most measurements, with 35% taking place on a Sunday, 23% on Saturday and 14% on Friday. Data to March 2022 were considered in the present analysis.

Stream gauge data is very limited or not existent for most of the monitored streams, which is typical for most rural catchments. A model for the relative annual variation of stream discharge was approximated using monthly precipitation data from the Radcliff Observatory from 1970 to 2021 (Burt & Burt, 2019). Seasonal dynamics in PO_4_ concentrations were then compared to expected seasonal changes in stream discharge to understand if pollution loads were continuous or event-driven. Continuous sources were expected to be sensitive to dilution from changes in stream discharge, while event-driven sources were expected to be more sensitive to precipitation frequency and intensity. Event duration monitoring data on the frequency and timing of event-driven pollution events from all the STWs in the catchment were supplied by Thames Water. Comparing event (spills) duration monitoring data with the PO_4_ concentrations measured by citizen scientists monitoring upstream and downstream locations of STWs provided additional information on event-driven conditions, with respect to continuous (chronic) emissions of wastewater without phosphorus removal mechanisms.

Two phosphate concentration limits were considered when evaluating PO_4_ concentrations. However, it should be noted that rivers have a characteristic baseline phosphate concentration, with headwater streams typically lower than concentrations in lowland rivers. Expressed as phosphorus phosphate, a common guideline for ecologically impacted lowland rivers is 0.10 mg PO_4_/L (Mainstone & Parr, 2002), although headwater streams can start to become impaired at concentrations above 0.05 mg PO_4_/L (Jarvie et al., 2018). It should be noted that concentration limits in streams may be higher in waterbodies with high alkalinity. It should be noted that the limit of 0.10 mg/L PO_4_ is aligned with the Water Framework Directive and typical for lowland waterbodies in the UK (Jarvie et al., 2018). The limits are intended as a guideline to reduce the risk of harmful algal blooms (e.g. cyanobacteria) as well as excessive algal and epiphytic algae growth with related impacts on biodiversity, dissolved oxygen and potable water sources.

To assess long-term changes in nutrient dynamics, EA and FWW data for sites with at least 100 measurements were used to estimate annual medians (a) over the last 5 decades and (b) between 2016 and present. A Mann–Kendall approach (Kendall, 1975; Ma et al., 2023) was used to assess the presence of a monotonic decrease (or increase) of PO_4_ over time in each monitoring location using annual median concentrations. The approach allows for linear or nonlinear trends and assumes that there are no seasonality, no autocorrelation and the absence of covariates. These were met by using annual medians to remove seasonality and reduce the influence of autocorrelation between monthly datasets. The slope of the annual change in PO_4_ concentrations was estimated using Sen’s slope method, which is more resistant to outliers and non-normal distributions than a least squares regression. Only years with at least 6 measurements per year were used in the analysis to ensure representativeness of the data. Each annual median concentration was considered representative of the conditions of each river for that year and used in the determination of Sen’s slope and the change per year in PO_4_ concentrations, considering the lower and upper bounds for each estimated slope. All data were tested with an alpha level of significance of 0.05, while alpha for multiple comparisons was corrected considering a Bonferroni correction. Sen’s slope (rate of change) and the most recent median concentration of PO_4_ for each site were used to estimate the years required for each site to reach concentration limits of 0.1 mg/L and 0.05 mg/L, assuming a linear trend in the catchment with continued improvements in in-stream conditions that influence river PO_4_ concentrations into the future. It should be noted that, while the overall trend is clear, the estimated rate of change per year is approximate, with lower and upper bounds, and as it assumes a linear trend where annual trends in median concentrations were not linear.

Seasonal dynamics of PO_4_ concentrations were determined by combining FWW and EA data for individual monitoring sites according to data availability, with equal weights given to both datasets for the determination of monthly median concentrations. The monthly dynamics of PO_4_ medians were used to classify each river or stream into three potential categories based on expected river discharge in the temperate climate zone of south England: low water PO_4_ maxima (June to September), high water PO_4_ maxima (November to March) and no clear seasonal dynamic. Each site was classified by its dominant seasonal dynamics in PO_4_ concentrations, based on whether the differences between low water median concentrations and high water median concentrations were statistically significant (*p* < 0.05, independent *T*-test of the low and high water median concentration).

An analysis of irregular PO_4_ concentrations for each site was made by using the irregular component of the 12-month moving average for each site. The difference between the actual measurement less the sum of the moving average and monthly average was considered to be the remaining irregular component. This irregular component (sum of the monthly irregular component medians) was then compared to the sum of the monthly median PO_4_ concentrations to identify sites with an elevated number of irregular events. Sites with a higher irregular component were those with more frequent or more intense periods with elevated PO_4_ concentrations, compared to sites with seasonal or inter-annual trends determined by the moving average. It should be noted that by using monthly median values, single extreme concentrations are not considered. Furthermore, this approach to temporal decomposition requires that the dataset is particularly long, as initial and final observations cannot be used in determining the moving average and also assumes that there are no other internal cycles beyond seasonality present in the data.

## Results

### Long-term trends

The sites for which long-term EA monitoring data exist cover a range of waterbodies and water quality conditions, with median concentrations of PO_4_ from less than 0.01 to nearly 3.00 mg/L (Table S1). Considering the most recent data (from 2016), a total of 67% of the sites had median concentrations above 0.1 mg/L PO_4_, and 80% had median concentrations above 0.05 mg/L PO_4_.

Combined data from EA and FWW monitoring showed that PO_4_ trends could be classified in two categories: sites where the PO_4_ concentrations displayed a negative (decreasing) trend over time and sites that showed no change (or a non-significant change) over time. A significant change was associated to a rate of decrease of at least 0.01 mg/L/year in PO_4_. For example, the ‘Four Shire Stream at Common Bridge’ shows a clear decrease in PO_4_ concentrations between 1989 and 2019, with a rate of change of 0.08 mg/L PO_4_ per year. An opposing example is the ‘Littlestock Brook above Evenlode at Shipton under Wychwood’, which showed no significant trend in PO_4_ over time (*p* > 0.05), suggesting that the concentrations did not have a monotonic decrease over the period of monitoring, considering both EA and FWW measurements in this site.

It should be noted that monitoring events are single events, typically monthly for FWW and every 2 months for EA monitoring. Each monitoring event is assumed to be representative of river conditions during the period of monitoring. Monitoring was performed during the daytime in both EA and FWW datasets and typically during the weekdays by the EA and on weekends by FWW citizen scientists. No step change in the overall concentrations of PO_4_ was observed over the period of available data.

Overall, six monitoring sites had a significant and consistent decrease in yearly median PO_4_ concentrations over the period of measurement. However, PO_4_ concentrations on the main Evenlode, as well as six streams and brooks, remain elevated and showed no decrease in PO_4_ concentrations over the last 5 decades. In several waterbodies, Bledington Brook and Glyme River, concentrations were initially and continue to remain low, with no temporal trend.

### Seasonality of PO_4_ concentrations

**Fig. 3:**
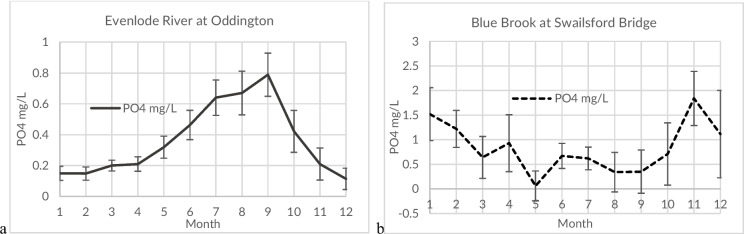
Measured median monthly and median standard error of phosphorus phosphate (PO4) concentrations in the **a** Evenlode River at Oddington (*n* = 315, 31 years) and **b** Blue Brook (downstream of a sewage treatment works) (*n* = 251, 25 years) determined by the national regulatory agency (Environment Agency)

The overall seasonality of the main Evenlode river generally followed a summer (low water) maximum, with the lowest concentrations occurring during the winter (high water) months. Many of the smaller streams and brooks also displayed a summer maximum, while two sites, including the Blue Brook, showed high water maxima, during periods of more elevated precipitation (Fig. 3). Of the six main Evenlode river monitoring sites with more than 100 monitoring events, only Moreton-in-the-Marsh showed no summertime maximum. This same site also had the lowest PO_4_ concentration during this period. Several sites, typically those with low overall concentrations, did not have any dominant maximum (Table S2).

### Seasonality of sites with suspected pollution sources

**Fig. 4:**
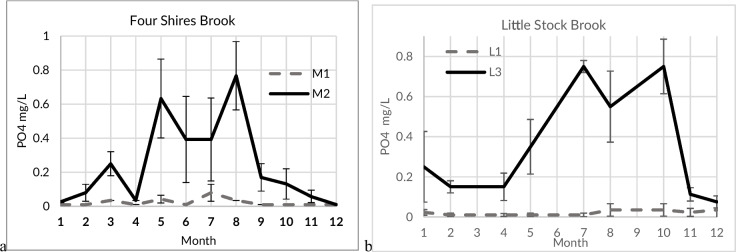
Seasonal dynamics of median monthly of phosphorus phosphate (PO4) measured by citizen scientists (FWW) between 2019 and 2021 upstream (dotted line) and downstream (solid line) of the sewage treatment works located in the **a** Four Shires Brook (*n* = 54, 2 years) and **b** Littlestock Brook (*n* = 131, 3 years)

Measurements (FWW) made by citizen scientists upstream and downstream of suspected pollution sources provided comparative information on the relative impact of active emission of PO_4_ into the receiving waters. The suspected pollution sources selected across the catchment were either STW or an STW with nearby agricultural or livestock activities. In all cases, sites located upstream of those suspected pollution sources did not show a clear seasonal dynamic, while seasonal dynamics at downstream sites were observed in several cases. Furthermore, monthly PO_4_ concentrations on Four Shires Brook, Hanborough Stream, Littlestock Brook, Blue Brook and Mill Stream (Fig. 4) were low in both upstream and downstream sites in December and January, while concentrations in downstream sites increased an order of magnitude between May and September. In these sites, there was no correlation between concentrations measured upstream and downstream (*p* > 0.05), suggesting that downstream concentration was not strongly influenced by upstream concentrations for most of the year. On the other hand, sites monitored by citizen scientists on the Dorn and Glyme rivers and on the Chadlington Brook showed significant correlations (*p* < 0.02) between upstream and downstream sites and no seasonal dynamics.

### Irregular behaviour of PO_4_ concentrations

**Fig. 5:**
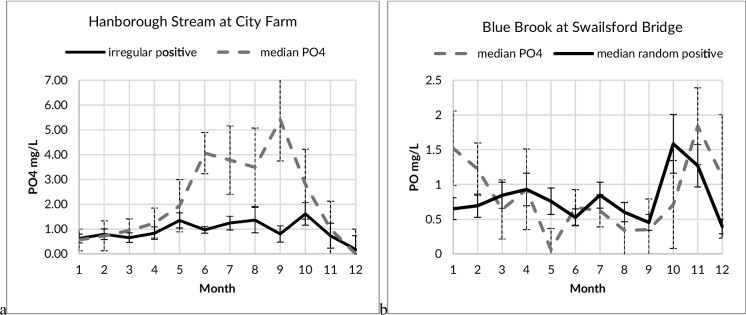
Seasonal dynamics of median monthly phosphorus phosphate (PO4) and the median of the monthly irregular component of PO4 concentrations with standard errors, based on a temporal decomposition at **a** Hanborough Stream (*n* = 210, 22 years) and **b** Blue Brook (*n* = 251, 25 years) using monitoring data from the national regulatory agency (Environment Agency)

The dynamics and relative intensity of the irregular maxima in PO_4_ concentrations, with respect to the seasonal or inter-annual trends, were explored to identify areas with anomalous pollution loads. Increases in the relative importance of the irregular components, with respect to the season components, suggested the presence of secondary drivers of PO_4_ dynamics. For example (Fig. 5 a), the irregular component (positive values) for Hanborough Stream shows no seasonal dynamic and is much lower than that of the median PO_4_ concentrations for this site, even though this site received discharges from an STW known for an elevated frequency of spills (Hammond et al., 2021). On the other hand, monitoring at Blue Brook at Swailsford Bridge (Fig. 5 b) has an irregular component that show clear seasonal dynamics, with higher concentration anomalies occurring in the high water months, similar to STW spill dynamics observed in 2021 (Table 1). In Cornwell Brook (not shown), the largest anomaly component occurs in the autumn, after the summer maximum of PO_4_ concentrations. Additional PO_4_ sources upstream of this site include road discharge, residential discharge and agriculture runoff. In general, sites with lower concentrations had the lowest relative irregular component.

**Table 1:** Anomaly analysis using temporal decomposition of PO_4_ concentrations in monitoring sites in the Evenlode catchment, showing the ratio of the irregular component with respect to the monthly median

Site	Number of measurements	Median mg/L PO_4_	% Irregular/monthly median
Bledington Brook above Evenlode	261	0.05	2.19
Blue Brook at Swailsford Bridge	286	1.04	1.04
Chadlington Stream above Evenlode	166	0.15	0.46
Cornwell Brook at Kingham	326	0.29	1.00
Dorn Above Glyme at Milford Bridge, Wootton	301	0.13	0.32
Evenlode at B4449, Cassington	550	0.18	0.40
Evenlode at Moreton In Marsh	175	0.09	NA
Evenlode at Oddington	418	0.32	0.40
Evenlode at Shipton Under Wychwood	381	0.25	0.37
Evenlode below Ashford Bridge	243	0.24	0.31
Evenlode at Coldicote Farm, Moreton in Marsh	106	0.12	0.27
Four Shire Stream at Common Bridge	259	1.89	0.51
Four Shire Stream just above Moreton in Marsh	131	0.10	NA
Glyme at A44, Woodstock	430	0.11	0.45
Glyme at Old Chalford	490	0.00	NA
Glyme at Wootton	337	0.00	NA
Hanborough Stream at City Farm	282	2.90	0.43
Heythrop Stream at Enstone	125	0.25	0.51
Little Compton Stream near Moreton In Marsh	234	0.16	0.51
Littlestock Brook at Shipton Under Wychwood	281	0.79	0.69

### Event duration monitoring data

STW Event Duration Monitoring data provided by Thames Water were compared to precipitation data. Spill events, both in number and in duration were more elevated during periods of high precipitation, in particular during the months of December, January, February and May, linked also to groundwater infiltration. Some STWs (e.g. Milton under Wychwood) had a higher spill frequency and longer duration than others.

Approximately half of the STWs have upstream monitoring sites, usually by citizen scientists, typically within 1 km upstream of the discharge point. In a similar manner, half of the STWs have downstream monitoring sites, usually within 1 km by both EA and citizen scientists. Combining data from citizen science monitoring upstream and downstream of STWs with event duration data and stream discharge estimates, the relative impact of continuous (chronic) emissions from STWs (point emission sources) to those from event-based spills was evidenced. One example is Four Shires Brook, where measurements were made by citizen scientists both upstream and downstream of the Moreton in Marsh STW. As expected, small summertime spills have a larger impact on stream PO_4_ concentrations compared to those that occur when river flow rates are expected to be higher. It should be noted that livestock activities also occur between the upstream and downstream locations.

## Discussion

**Fig. 6:**
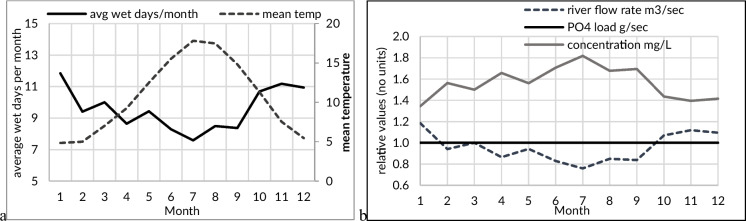
**a** Seasonal dynamics of precipitation and temperature from the Radcliff Observatory, **b** hypothetical median monthly phosphorus phosphate (PO4) dynamics under a constant load and a summertime decrease in river flow rate

Industrial, agricultural activities or STWs without tertiary treatment can release a relatively constant load of PO_4_ into receiving waters (e.g. rivers). In periods of elevated stream/river discharge, assuming a constant input to the river, PO_4_ concentrations are lower due to a higher dilution (Fig. 6). In periods of low flow, typically summer in temperate climates, the opposite occurs, with PO_4_ maxima occurring. It should be noted that internal sources and river geochemistry can also drive seasonal PO_4_ concentration dynamics, with studies showing the mobilisation of phosphorus in the summer from sediments with elevated phosphorus in the presence of iron can also occur, in conditions of low dissolved oxygen (< 4 mg L^−1^) (Smolders et al., 2017).

Importantly, the highest biological demand for phosphorus is also in this period, presenting a potential PO_4_ sink^6^. However, summer peaks in PO_4_ favour increased algal growth both within the water column and as periphyton, with direct consequences on macrophyte community. This is particularly impactful on the submerged macrophyte community, where periphyton growth reduces available light resulting in a loss of submerged macrophytes and increased dominance of emergent macrophytes. Citizen scientists have reported the loss of traditional macrophytes in many brooks and streams in the Evenlode catchment (personal communication), but quantitative data are lacking.

Seasonal PO_4_ dynamics that more closely follow precipitation dynamics indicate more episodic PO_4_ loads and can be caused by point as well as diffuse pollution sources (Moshi et al., 2022). These can include agricultural runoff, combined sewage overflows (spills) and stored phosphorus in river sediments, all of which can release more PO_4_ during periods of elevated precipitation. Other episodic PO_4_ sources related to precipitation include leaking or overflowing septic systems from rural houses and road runoff.

Using the observed overall rate of change (Sen’s slope) to estimate the years required for each site to reach concentration limits of 0.1 mg/L and 0.05 mg/L showed that nearly half of the sites are below or will be below the limits of 0.10 mg/L in the near future (Table S3). It should be noted that a continued and relatively linear change is unlikely to occur at lower concentrations as legacy phosphorus will become increasingly important. However, many of the smaller streams and brooks (Chadlington Stream, Dorn above Glyme, Four Shire Stream and Littlestock Brook) have shown limited or no reduction in PO_4_ and therefore are not expected to reach either limit (NA). Most importantly, data from the main river sites in Moreton in Marsh, Oddington and Cassington (opposite sides of the catchment) present similar conditions of limited or no improvement.

Many of these same sites present a strong irregular component, including Bledington Brook, Blue Brook, Cornwell Brook, Four Shire Stream, Littlestock Brook and Little Compton Stream as well as the Evenlode at Moreton in Marsh. Most of these are located downstream from STWs, suggesting the high degree of anomalies may be associated to STW related spills. However, anomalies in Bledington Brook and Cornwell Brook may also be related to potential intermittent PO_4_ sources from agriculture or residential runoff. It should be noted that the high anomaly values of two of the Glyme sites occur where PO_4_ concentrations are low (near or at the detection limit) and should not be considered as true anomalies. Most important are the higher irregular components in waterbodies where PO_4_ concentrations are already elevated, indicating concentration maxima that follow neither the expected seasonal behaviour nor the median concentrations of that year.

## Conclusions

The cooperation between a wide range of stakeholders in Evenlode presents an important opportunity to share knowledge and work towards solutions. Within the same project, additional stakeholders, including farmers and educational institutes, are also involved in complementary workstreams. The cooperation between monitoring partners, the Environment Agency, Thames Water and citizen scientists has allowed for the creation of an online dataset, which is updated monthly and used to inform all stakeholders through regular newsletters and reports. These activities require collaboration of all partners through regular meetings and consultation, where individual expectations and aims are recognised. Similar cooperation has also supported the exploration of potential mitigation activities in the catchment (Robotham et al., 2023), showing the opportunities of nature-based solutions to reduce sediment and phosphorus transport.

The results of these combined data provide an improved picture of the overall conditions of the Evenlode catchment. While long-term trends in several waterbodies show improvements in PO_4_ concentrations, many smaller streams and brooks remain heavily impacted. Most of these, as well as the main Evenlode river, fail to reach good chemical status in terms of the WFD, clearly showing the need for interventions to reduce phosphorus load. It should be noted that, even after mitigation of phosphate sources, improvements to river conditions may require years or even decades. Indirect improvements (e.g. increased biodiversity) following mitigation (e.g. reduced nutrient loads) depend on multiple and related factors, all influenced by modifications to stream hydrology, geochemistry and geomorphology, as well as riparian and in-stream vegetation and legacy pollutant loads (Goyette et al., 2018). Recovery may follow a nonlinear trajectory if baseline conditions (riparian vegetation, sediment conditions, river morphology) have changed as a direct or indirect result of high PO_4_ load, requiring lower PO_4_ concentrations than those of the original unimpacted river (Lorenz et al., 2018; Wurtsbaugh et al., 2019).

Pollution source monitoring by citizen scientists in the Evenlode catchment provided an opportunity to fill gaps in regulatory monitoring as well as prioritise management actions. Benefits include an improved temporal resolution for the identification of the seasonal dynamics of stream conditions, in relation to continuous or even-driven nutrient sources. Monthly or near monthly monitoring by citizen scientists, compared to bimonthly or quarterly EA monitoring, improves the identification of nutrient concentration dynamics in relation to seasonal changes in precipitation and temperature. It should be noted that citizen scientist monitoring has inherent biases, with most measurements occurring on the weekend and only during the daytime. Monthly spot monitoring, in general, is less informative than continuous monitoring to capture diurnal and weekly variations but can be used to show seasonal trends and the presence of anomalous conditions. Large-scale (high spatial resolution) spot sampling by citizen scientists, for example, in blitz-type monitoring events, can also be used to inform strategies for continuous monitoring or high-frequency spot sampling. In general, the increased coverage of low-cost measurements is providing important new information on spatially and temporally variable pollution dynamics and not only for water pollution monitoring (Frederickson et al., 2022).

Spatially, the approach taken by citizen scientists in the Evenlode presents a number of innovations but also challenges often associated to citizen science. Site selection by citizen scientists is limited to areas with relatively easy access and typically in locations that they regularly frequent (for work, home or hobby). However, the approach taken by the citizen scientists in the Evenlode, to monitor in multiple locations around potential pollution sources, fills important information gaps with respect to the EA focus on the central river and downstream sites. This approach provided important evidence of the relative importance of seasonal impact of pollution sources, in this case, STWs.

The hypothesis-based approach taken by citizen scientists in the Evenlode showed the power of citizen science to prove new insights. While not all the suspected pollution sources proved to be active sources, many showed significant increases in PO_4_ concentrations in downstream sites, particularly during the summer, suggesting an active source whose impact is further increased by reduced dilution rates. While increased PO_4_ release from STWs in the absence of tertiary treatment is not unexpected, the data obtained by citizen scientists allows for the prioritisation of mitigation activities in streams with limited dilution capacity. Given the pressure on water companies to install phosphate removal mechanisms, the Partnership identified these smaller waterbodies as priority while still addressing capacity issues. The actual introduction of phosphorus removal mechanisms to the smaller STWs in the catchment will depend on multiple factors, outside the Partnership. In those stream and river sites showing that showed event-based concentration dynamics, either intermittent or precipitation-driven, partners have focused efforts to identify these sources, for example, using Outfall Safari, as a first step to managing them. A similar approach to using citizen scientist monitoring for other pollution sources, nitrate, suspended sediment or microbiological conditions can also complement regulatory monitoring as well as support local prioritisation of mitigation actions to reduce agricultural impacts on receiving rivers and lakes.

In the present study, we have not assigned different weights to regulatory versus citizen scientist monitored concentrations based on the increased uncertainty of individual measurements made by citizen scientists. However, in a similar manner to that being suggested for SDG indicator reporting (personal communication), combining data from multiple sources and assigning different levels of reliability could be used to incorporate citizen scientist acquired data into national and international databases. The use of multiple data sources requires an understanding of their individual biases and uncertainties.

The complementary of citizen scientist and regulatory monitoring required a collaboration between partners, both for data sharing and for optimising efforts to utilise the information acquired. The availability of long-term monitoring by regulatory agencies, as well as increased frequency and coverage provided by trained citizen scientists, increased the possibility to identify potential sources and represents a novel participatory approach to rural basin nutrient management. This study shows the opportunity for regulatory agencies to incorporate robust citizen scientist monitoring into their overall monitoring and management schemes.

### Supplementary Information

Below is the link to the electronic supplementary material.

Supplementary file1 (DOCX 56.5 KB)

## Data Availability

Citizen science data are open data, available on the FWW website https://www.freshwaterwatch.org/. All regulatory data are available on the EA website https://environment.data.gov.uk/catchment-planning/OperationalCatchment/3181. Thames Water data are available from the corresponding author on reasonable request.
