# Supplementary material for: Citizen scientists filling knowledge gaps of phosphate pollution dynamics in rural areas
Source: Environ Monit Assess. 2024 Jan 31;196(2):220. doi: 10.1007/s10661-024-12389-5 (PMC10827960; doi:10.1007/s10661-024-12389-5)
Supplement: Supplementary file 1 — Supplementary file1 (DOCX 56.5 KB) [file 10661_2024_12389_MOESM1_ESM.docx]

Supplemental information

*Table S1. Environment Agency monitoring of PO_4_ concentrations in different waterbodies in the Evenlode catchment, with percentage of monitoring events above 0.01 mg/L and 0.05 mg/L since 2016.*

| **Site** | **Median mg/L** PO_4_ | **Median from 2016**  **mg/L** PO_4_ | **From 2016 %>0.1 mg/L** | **From 2016 %>0.05 mg/L** |
| --- | --- | --- | --- | --- |
| Bledington Brook above Evenlode | 0.05 | 0.04 | 4% | 33% |
| Blue Brook at Swailsford Bridge | 1.04 |  |  |  |
| Chadlington Stream above Evenlode | 0.15 |  |  |  |
| Cornwell Brook at Kingham | 0.29 | 0.16 | 88% | 100% |
| Dorn Above Glyme at Milford Bridge, Wootton | 0.13 | 0.10 | 56% | 81% |
| Evenlode at B4449, Cassington | 0.18 | 0.12 | 72% | 94% |
| Evenlode at Moreton In Marsh | 0.09 |  |  |  |
| Evenlode at Oddington | 0.32 | 0.21 | 100% | 100% |
| Evenlode at Shipton Under Wychwood | 0.25 | 0.09 | 83% | 100% |
| Evenlode below Ashford Bridge | 0.24 | 0.10 | 76% | 100% |
| Evenlode at Coldicote Farm, Moreton in Marsh | 0.12 | 0.11 | 65% | 100% |
| Four Shire Stream at Common Bridge | 1.89 | 0.51 | 100% | 100% |
| Four Shire Stream just above Moreton in Marsh | 0.10 |  |  |  |
| Glyme at A44, Woodstock | 0.11 | 0.06 | 57% | 93% |
| Glyme at Old Chalford | <0.01 | <0.01 | 0% | 0% |
| Glyme at Wootton | <0.01 | <0.01 | 0% | 0% |
| Hanborough Stream at City Farm | 2.90 | 0.71 | 100% | 100% |
| Heythrop Stream at Enstone | 0.25 |  |  |  |
| Little Compton Stream near Moreton In Marsh | 0.16 | 0.10 | 58% | 92% |
| Littlestock Brook at Shipton Under Wychwood | 0.79 | 0.14 | 100% | 100% |

*Table S2: Seasonal behaviour of monitoring sites with at least 100 measurements in the Evenlode catchment.*

| **Site** | **Seasonal behaviour** |
| --- | --- |
| Bledington Brook above Evenlode | Low water maxima |
| Blue Brook at Swailsford Bridge | High water maxima |
| Chadlington Stream above Evenlode | High water maxima |
| Cornwell Brook at Kingham | Low water maxima |
| Dorn Above Glyme at Milford Bridge, Wootton | Low water maxima |
| Evenlode at B4449, Cassington | Low water maxima |
| Evenlode at Moreton In Marsh | Limited seasonal dynamic |
| Evenlode at Oddington | Low water maxima |
| Evenlode at Shipton Under Wychwood | Low water maxima |
| Evenlode below Ashford Bridge | Low water maxima |
| Evenlode at Coldicote Farm, Moreton in Marsh | Low water maxima |
| Four Shire Stream at Common Bridge | Low water maxima |
| Four Shire Stream just above Moreton in Marsh | Limited seasonal dynamic |
| Glyme at A44, Woodstock | Low water maxima |
| Glyme at Old Chalford | No clear seasonal dynamic |
| Glyme at Wootton | No clear seasonal dynamic |
| Hanborough Stream at City Farm | Low water maxima |
| Heythrop Stream at Enstone | Low water maxima |
| Little Compton Stream near Moreton In Marsh | Low water maxima |
| Littlestock Brook at Shipton Under Wychwood | Low water maxima |

*Table S3. Estimated rate of change of PO_4_ concentrations in Environment Agency monitoring sites in the Evenlode, with estimated year of reaching* PO_4_ *concentrations of 0.10 mg/Land 0.05 mg/L.*

| **Site** | **Most recent year of monitoring** | **Median from 2016** | **Predicted year @ 0.1 mg/L** | **Predicted year @ 0.05 mg/L** |
| --- | --- | --- | --- | --- |
| Bledington Brook above Evenlode | 2020 | 0.04 | 1995 | 1998 |
| Blue Brook at Swailsford Bridge | 2004 | NA | 2010 | 2011 |
| Chadlington Stream above Evenlode | 2004 | NA | NA | NA |
| Cornwell Brook at Kingham | 2020 | 0.16 | 2022 | 2026 |
| Dorn Above Glyme at Milford Bridge, Wootton | 2020 | 0.10 | 2018 | 2035 |
| Evenlode at B4449, Cassington | 2021 | 0.12 | 2025 | 2041 |
| Evenlode at Moreton In Marsh | 2004 | NA | NA | NA |
| Evenlode at Oddington | 2021 | 0.21 | 2028 | 2033 |
| Evenlode at Shipton Under Wychwood | 2020 | 0.09 | 2017 | 2020 |
| Evenlode below Ashford Bridge | 2021 | 0.10 | 2018 | 2014 |
| Evenlode at Coldicote Farm, Moreton in Marsh | 2021 | 0.11 | NA | NA |
| Four Shire Stream at Common Bridge | 2021 | 0.51 | 2023 | 2024 |
| Four Shire Stream just above Moreton in Marsh | 2002 | NA | NA | NA |
| Glyme at A44, Woodstock | 2021 | 0.06 | 2008 | 2021 |
| Glyme at Old Chalford | 2019 | 0.00 | 1990 | 1990 |
| Glyme at Wootton | 2017 | 0.00 | 1990 | 1990 |
| Hanborough Stream at City Farm | 2020 | 0.71 | 2022 | 2022 |
| Heythrop Stream at Enstone | 2004 |  | NA | NA |
| Little Compton Stream near Moreton In Marsh | 2021 | 0.10 | 2018 | 2026 |
| Littlestock Brook at Shipton Under Wychwood | 2020 | NA | NA | NA |
